# Apoptotic and Inhibitory Effects on Cell Proliferation of Hepatocellular Carcinoma HepG2 Cells by Methanol Leaf Extract of *Costus speciosus*

**DOI:** 10.1155/2014/637098

**Published:** 2014-04-10

**Authors:** Sandhya V. G. Nair, Menik Hettihewa, H. P. Vasantha Rupasinghe

**Affiliations:** ^1^Department of Environmental Sciences, Faculty of Agriculture, Dalhousie University, Truro, NS, Canada B2N 5E3; ^2^Department of Pharmacology, Faculty of Medicine, University of Ruhuna, Karapitiya, Sri Lanka

## Abstract

*Costus speciosus* is a medicinal plant commonly known as wild ginger distributed in South and Southeast Asian countries. Leaves of this plant are used for ayurvedic treatment regimes in malignancies and mental illness. Rhizome extract from the plant is used to treat malignancies, pneumonia, urinary disorders, jaundice, rheumatism, and diabetes. The goal of this study was to investigate the effects of methanol extract of leaves of *C. speciosus* on the growth of human hepatocellular carcinoma (HepG2) cells and understand possible mechanisms of its action. Viability of HepG2 cells were measured by MTS assay after 24 h and 48 h treatment with extracts of 1, 10, 50, 100, and 200 **μ**g/mL concentrations. Cell cycle analysis and apoptosis were evaluated by flow cytometry and caspase-3 induction. HepG2 cells treated with 100 **μ**g/mL methanol leaf extract for 24 h displayed a significant reduction in cell viability (*P* ≤ 0.05). The methanol extract perturbed cell cycle progression, modulated cell cycle and regulated, signal molecules were involved in induction of apoptosis in HepG2 cells. Our findings indicate that phytochemicals of leaves of *C. speciosus* shows potential for natural therapeutic product development for hepatocellular carcinoma. This is the first report to demonstrate *in vitro* anticancer activity of leaf extract of *C. speciosus* in relation to liver cancer.

## 1. Introduction

Hepatocellular carcinoma, the predominant primary liver cancer in most countries, is the fifth most frequent cancer in the world [1]. Several etiological factors have been identified including chronic infection with hepatitis B virus (HBV) or hepatitis C virus (HCV), prolonged exposure to aflatoxin B1 [2], liver cirrhosis due to alcohol abuse, and nonalcoholic fatty liver [3]. Patients with early disease are often asymptomatic and consequently late diagnosis makes the disease untreatable. Therapeutic choices are surgical resection [4], transplantation [5], and ablation in which the survival rate is known to be less than 25% [6]. The only noncurative treatments that improve survival are transarterial chemoembolization [7] and chemotherapy with sorafenib (Nexavar), a MAP kinase pathway inhibitor [8]. Sorafenib, a drug for prolonging the survival of hepatocellular carcinoma patients, is generally well tolerated but diverse results in terms of the safety and the efficacy have been obtained: some patients show severe toxicities such as hand and foot skin reactions, diarrhea, hyperbilirubinemia, and fatigue; some patients show very short survival [9]. Therefore, development of an effective cancer chemotherapeutic agent with fewer side effects is an urgent need for management of hepatocellular carcinoma. The investigations of the efficacy of plant-based drugs have received growing attention due to their no or minimum side effects.

Traditional medicinal plants that have less or no toxicity and have chemotherapeutic and chemopreventive activities are currently being investigated for their possible clinical application in cancer treatment and prevention [10].* Costus speciosus* Koen (also called “Thebu,” Crape, or wild ginger) is a medicinal perennial herb found in the south and southeast Asian countries. It has been widely used in ayurveda system of medicine to treat diverse ailments [11]. Rhizome and roots (extracts) are useful as astringent, aphrodisiac, purgative, anthelmintic, depurative, febrifuge, and expectorant. Areal parts of this plant had been tested to reduce fever and to treat mental disorder [11]. Rhizome and root (extracts) have been extensively studied for numerous medicinal properties including antidiabetic [12], hypolipidemic [12], anticholinesterase, hepatoprotective, antistress, antioxidant, antibacterial, antifertility, anti-inflammatory, and antipyretic properties (Srivastava et al., 2011).

The major chemical constituents of* C. speciosus* are diosgenin, curcumin, and curcuminoids (Rani et al., 2012). The rhizome and roots also contain saponins, 5*α*-stigmasten-3b-ol, sitosterol-*β*-D-glucoside, dioscin, prosapogenins A and B of dioscin, gracillin, and quinines (Rani et al., 2012). Diosgenin is reported to induce apoptosis in human leukemia cells [13]. Curcumin and curcuminoids are well known for their chemotherapeutic property on solid cancer as well as leukemia [14].

To date, there is only one report of* C. speciosus* rhizome extract on human cancer cell lines which showed the* in vitro* inhibition of cell proliferation and induced apoptosis in cancer cell lines [15]. No studies of* C. speciosus* leaf extract were reported on liver cancer. Therefore, we have explored the antiproliferative effects of* C. speciosus* leaf extract on human hepatocellular carcinoma cells (HepG2) which could lead to future clinical trials for liver cancer patients.

## 2. Materials and Methods

### 2.1. Preparation of Methanol and Hexane Extracts of* C. speciosus* Leaves

Fresh* C. speciosus* leaves identified from Sri Lanka were freeze dried and ground into a fine powder before shipping to Canada. Methanol extracts were prepared by treating the fine powder (0.5 g) with 100% methanol (20 mL) and sonicated for 20 min at room temperature. After centrifugation at 3000 rpm for 10 min, the supernatant was filtered through a 0.2 *μ*m nylon filter. The extract was evaporated to dryness and stored at −20°C. For preparation of hexane extract, the finely powdered* C. speciosus* leaves (1 g) were extracted with hexane and ethyl acetate (40 mL of hexane: 1 mL of ethyl acetate) and sonicated for 20 min at room temperature. The solvent was filtered through a 0.2 *μ*m filter and evaporated to dryness and stored at −20°C until used for* in vitro* assay. Dried methanol and hexane extract were dissolved separately in DMSO and diluted accordingly with culture medium for* in vitro *experiments.

### 2.2. Chemicals

Cell Titer 96 Aqueous One solution cell proliferation (MTS) assay (Promega, Madison, WI, USA) and caspase-3 colorimetric assay (Sigma-Aldrich, Mississauga, ON, Canada) kits were used for the study. Sterile dimethyl sulfoxide (DMSO) (ATCC, Rockville, USA), GFP-certified apoptosis/necrosis detection kit for flow cytometry from Enzo Life Sciences (Farmingdale, NY, USA), propidium iodide (Sigma-Aldrich, Mississauga, ON, Canada), and JC-1 (Cayman Chemicals, Ann Arbor, Michigan, USA) were purchased for the experiment.

### 2.3. Cell Lines and Culture Conditions

Human hepatocellular carcinoma cell line (HepG2), human acute monocytic leukemia cells (THP-1), and human normal lung cells (WI-38) were purchased from the American Type Culture Collection (ATCC, Rockville, MD, USA) and cultured as recommended by the ATCC. HepG2 and WI-38 cells were grown in Eagle's modified minimum essential media (EMEM) supplemented with 10% FBS (FBS; ATCC, Rockville, MD, USA) and 1% penicillin-streptomycin (ATCC, Rockville, MD, USA). THP-1 cells were cultured in RPMI-1640 media supplemented 0.05 mM 2-mercaptoethanol and 10% fetal bovine serum to a final concentration of 10%. Cells were maintained at 37°C in an incubator under 5% CO_2_/95% air atmosphere at above 85% relative humidity constantly. The cells were cultured (about 80% of confluents) once a week in T-75 flasks and the media were changed one additional time a week. Experiments were conducted using cells with less than 20 passages. Cells were counted using a haemocytometer (Bright-Line Hemacytometer, Sigma-Aldrich (Mississauga, ON, Canada) and were plated according to the number of cells for each experiment in 6-, 24-, or 96-well format for 24 h prior to addition of test compounds. All the test samples were solubilized in sterile filtered DMSO (<0.5% in the culture medium) prior to addition to the culture media. Control cells were also run in parallel and subjected to the same changes in media with <0.5% DMSO.

### 2.4. Cell Proliferation Assay

HepG2, THP-1, and WI-38 cells (1 × 10^4^ cells/100 *μ*L/well) were seeded in a sterile flat bottom 96-well plate (BD Biosciences, Mississauga, ON, Canada) and stabilized by incubation for 24 h at 37°C in a humidified incubator containing 5% CO_2_ (VWR, Mississauga, ON, Canada). The methanol and hexane extracts of* C. speciosus* leaves or sorafenib were prepared in media and 100 *μ*L of each treatment was added to each well, each treatment in six replications. Thereby, cells were exposed to various concentrations (1, 10, 50, 100, and 200 *μ*g/mL) of each treatment. Controls consist of cells with media containing DMSO (<0.5%) and blank wells contained media with no cells. After 24 and 48 h of test compound incubation, 20 *μ*L of the MTS reagent in combination with the electron coupling agent phenazine methosulfate was added to the wells and cells were incubated in a humidified CO_2_ incubator for 3 h. Absorbance at 490 nm (OD490) was monitored with a plate reader (FLUOstar Optima, BMG Labtech, Durham, NC, USA) to obtain the number of viable cells relative to the control population. Percentage of viability in the test compound treated cells are expressed as percentage compared to control (<0.5% DMSO). Data are expressed as mean values ± SD and obtained from three different experiments against each cell line (*n* = 6 per plate per time point).

### 2.5. Morphological Observation under Inverted Phase Contrast Microscope

HepG2 cells were equally seeded in 24-well flat bottom tissue culture treated plates (BD Biosciences) and then treated with 100 *μ*g/mL of the extracts, sorafenib, or DMSO (<0.5%) control. After 24 h of treatment, the morphology of HepG2 cells was observed under an inverted phase contrast microscope (Nikon Eclipse E 100, Nikon, ON, Canada) and images were captured at 400x magnification using Infinity digital microscopy camera (Lumenera corporation, ON, Canada).

### 2.6. Determination of Apoptosis/Necrosis by Flow Cytometry

HepG2 cells were treated with 100 *μ*g/mL extracts, sorafenib, or DMSO control (<0.5%) for 24 h. After treatment, cells were harvested, washed with PBS, and resuspended in 500 *μ*L of binding buffer containing 5 *μ*L Annexin V and 5 *μ*L 7-AAD and incubated in dark for 15 min according to the instructions of the manufacture (Enzo Life Sciences Inc., Farmingdale, NY, USA). After incubation, samples were immediately analyzed by flow cytometry (FACS Calibur, Beckman Coulter, Fullerton, Cam, USA) using a 488 nm laser.

### 2.7. Assay of Caspase-3 Activity

The activity of caspase-3 enzyme was measured using Caspase-Glo3/7 reagent kit purchased from Promega (Madison, WI, USA). HepG2 cells (1 × 10^4^ cells/well), grown in 96-well white plates, were treated either with 100 *μ*g/mL extract, sorafenib, or DMSO vehicle (as control). After 12 h and 24 h incubation in an 37°C/5% CO_2_ humdified incubator, 100 *μ*L of Caspase-Glo3/7 reagent was added to each well containing 100 *μ*L of blank, negative control cells, or treated cells in culture medium. After mixing the contents of wells using a plate shaker for 30 seconds, plates were incubated at room temperature for 2 h. Luminescence was measured using a flurostar optima microplate reader (BMG Labtech, NY, USA).

### 2.8. Measurement of Mitochondrial Membrane Potential (MMP)

Mitochondrial membrane potential, Δ*ψm*, is an important parameter of mitochondrial function used as an indicator of cell health. JC-1 is a lipophilic, cationic dye that can selectively enter into mitochondria and reversibly change color from green to red as the membrane potential increases. In healthy cells with high mitochondrial Δ*ψm*, JC-1 spontaneously forms complexes known as J-aggregates with intense red fluorescence. On the other hand, in apoptotic or unhealthy cells with low Δ*ψm*, JC-1 remains in the monomeric form, which shows only green fluorescence.

Briefly, HepG2 cells were seeded in a 96-well black plate at a density of 5 × 10^4^ cells/well (100 *μ*L) and incubated in a CO_2_ incubator for 24 h at 37°C. Cells were treated with 100 *μ*g/mL of extracts or sorafenib and control cells with vehicle (<0.5% DMSO) and incubated for 24 h. The staining solution JC-1 (Cayman Chemicals, Ann Arbor, Michigan, USA) was prepared with PBS and 5 *μ*M was added to each well. The cells were further incubated in a CO_2_ incubator at 37°C for 1 h. After washing the plate with PBS twice, the fluorescence was measured using an (FLUOstar Optima, BMG Labtech, Durham, NC, USA) at 535 nm for JC-1 monomers and at 590 nm for JC-1 aggregate. The cells were also observed under a fluorescence Zeiss Axiovert 200 m inverted microscope (Carl Zeiss, Toronto, ON, Canada) at magnification of ×40 with a filter set for JC-1 aggregates (Ex/Em: 540/570 nm) and JC-monomers (Ex/Em: 485/535 nm).

### 2.9. Cell Cycle Analysis

HepG2 cells (5 × 10^5^ cells) in a six-well plate were incubated (37°C, 5% CO_2_) with 100 *μ*g/mL extracts or sorafenib or DMSO vehicle (<0.5%) (as control) media for 24 h. Following trypsinization, cells were washed and centrifuged at 2000 ×g for 10 min and the pellet was resuspended in 0.5 mL PBS. Fixation was completed by adding 1.2 mL of 70% cold ethanol for 2 h. The fixed cells were washed with PBS and centrifuged at 2000 ×g for 10 min. After suspending cells in 0.3 mL PBS, 8 *μ*L of DNAase free RNAse (10 mg/mL) was added and incubated for 1 h. After adding 15 *μ*L of propidium iodide (0.5 mg/mL), cells were incubated at 4°C for 30 min. The cells were analyzed for cell cycle using flow cytometer FACS calibur (Beckman Coulter, Fullerton, CA, USA) with an excitation wavelength of 488 nm and emission at 670 nm. DNA content was determined by ModFit software (Verity Software House, Topsham, ME), which provided histograms to evaluate cell cycle distribution.

### 2.10. Statistical Analyses

GraphPad Prism (version 6 for Windows, GraphPad Software Inc., San Diego, USA) was employed to produce dose-response curves by performing nonlinear regression analysis. For each concentration, percent cell viability values were calculated and cell viability was plotted versus drug concentrations in the logarithmic scale. EC_50_ values were determined using a four-parameter dose response (variable slope) equation in GraphPad Prism Statistical analysis was performed using Statistical Analysis System (SAS, Version 9.2). One-way ANOVA with Tukey's post hoc comparisons at *P* < 0.001 was used for statistical comparisons. All data are presented as a mean value with its standard deviation indicated (Mean ± SD).

## 3. Results

### 3.1. Optimum Dose of* C. specious* Leaf Extracts to Inhibit HepG2 Cell Proliferation

We first sought to determine the optimum dose of the methanol and hexane extracts to inhibit the proliferation of the hepatocellular carcinoma cell line. HepG2 cells were treated with increasing concentrations of methanolic extract or hexane extract or sorafenib (1, 10, 50, 100, and 200 *μ*g/mL), and cell viability was assayed at 24 and 48 h after treatment. The methanol extract resulted in a dose- and time-dependent decrease in cell viability. The sensitivity of the methanol extract was more prominent in longer incubation time points and higher dosage. The hexane extract did not show any antiproliferative effect even at high concentration or later time point. Therefore, hexane extract was excluded from the further study (Table 1). Treatment with the methanol extract (100 *μ*g/mL) for 24 h decreased the cell viability of hepatocellular carcinoma HepG2 and leukemia THP-1 cells with EC_50_ value of 93.3 and 58.1 *μ*g/mL, respectively, which further decreased to 77.3 and 42.2 *μ*g/mL at 48 h compared to the control cells (Table 2).

To evaluate the specificity of methanol extract to cancer cells, effect of the extract on the viability of normal cells was quantified by cytotoxicity assay using normal human lung cells WI-38. The cells were treated with 200 *μ*g/mL and lower concentrations of methanol extract for 24 h and 48 h (Table 1). Methanol extract did not affect the viability of normal human lung cells with EC_50_ >100 *μ*g/mL and is more specific to cancer cell lines (Table 2). HepG2 cells were chosen as the representative cell line and the methanol extract of 100 *μ*g/mL for 24 h was selected for the subsequent experiments.

### 3.2. Microscopic Evaluation of Morphological Changes in HepG2 Cells

**Figure 1:**
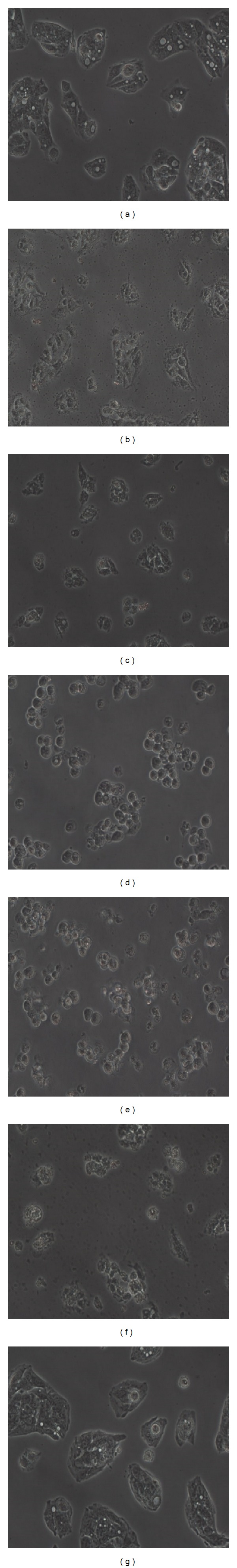
Inverted phase contrast microscopy showing methanolic extract of* Costus speciosus* leaves or sorafenib (positive control) induced changes of HepG2 cells. The control cells were well adhered, displaying the normal morphology of HepG2 cells. In contrast, majority of HepG2 cells treated with methanol extract at the concentration of 100 *μ*g/mL or more became round and shrunken and could not be affixed to the walls and floating in the medium (×400 magnification). (a) 50 *μ*g/mL methanol extract; (b) 100 *μ*g/mL methanol extract; (c) 200 *μ*g/mL methanol extract; (d) 50 *μ*g/mL sorafenib; (e) 100 *μ*g/mL sorafenib; (f) 200 *μ*g/mL sorafenib; (g) control.

To examine the effect of the methanol extract on cell morphology, HepG2 cells were treated with 50, 100, and 200 *μ*g/mL of the extract or sorafenib (positive control) for 24 h and morphological changes were observed by phase contrast microscopy. The images showed that the methanol extract induced severe morphological changes of cell death including rounding and shrinkage of cells in a dose-dependent manner. The pattern of cell death was similar to liver cancer drug sorafenib (positive control) at all concentrations (Figure 1).

### 3.3. The Methanol Extract Induces Apoptosis in HepG2 Cells

**Figure 2:**
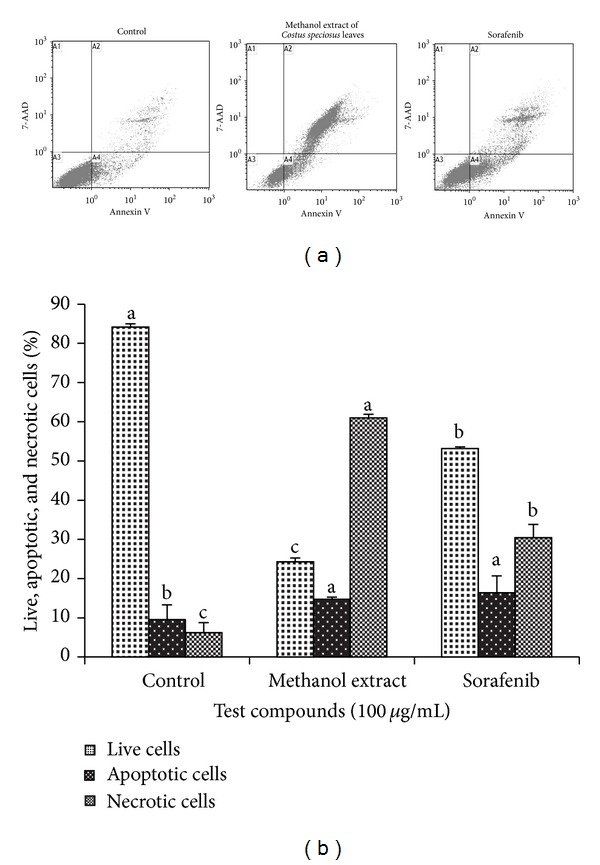
FACS analyses of Annexin V and 7-AAD staining. HepG2 cells were treated with methanolic extract of* Costus speciosus* leaves or sorafenib (100 *μ*g/mL) for 24 h. Lower right quadrant (D4) Annexin V positive/7-AAD negative cells denote early apoptosis cells and upper right quadrant (D2) Annexin V positive/7-AAD positive cells denote necrosis or late apoptotic cells. Images are the representative of three independent experiments.

The effects of the methanol extract or sorafenib on apoptosis were evaluated by using Annexin V/7-Aminoactinomycin D (7-AAD) staining through flow cytometry. Translocation of phosphatidylserine (PS) to the outer leaflet of cellular membrane is the key step in the early stages of apoptosis. Annexin V selectively binds to PS and helps to identify cells undergoing apoptosis. 7-AAD is a red fluorescent, live-cell impairment chemical compound that intercalates in double-stranded DNA, with a high affinity for GC-rich regions. When cells are double stained with Annexin V/7-AAD, the different populations of cells can be observed. The cells that do not stain with either Annexin V or 7-AAD are alive and reside in region A3; the cells that stain with only Annexin V are in the stage of early apoptosis and reside in region A4 while the cells that stain with both reagents are nonviable late apoptotic/necrotic cells and scatter in region A2 (Figure 2(a)). The percentages of apoptotic and necrotic cells on methanol extract treatment were 14.7 and 61%, respectively (Figure 2(b)). Flow cytometric analysis of apoptosis showed that the methanol extract induced apoptosis in HepG2 cells similarly to sorafenib suggesting that the methanol extract induced cell death was though early apoptosis.

### 3.4. The Methanol Extract Induces Caspase-3 Activation in HepG2 Cells

**Figure 3:**
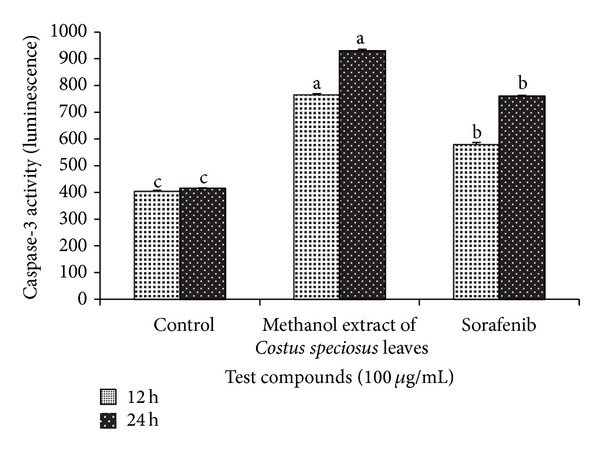
Effects of methanolic extract of* Costus speciosus* leaves in HepG2 cells. Caspase-3 activity significantly increased on treatment with methanol extract or sorafenib at a concentration of 100 *μ*g/mL for 24 h compared to control. Data are expressed as mean ± SD (*n* = 3). Columns not sharing the same superscript letter differ significantly (*P* < 0.05).

Mitochondrial dependent apoptosis is initiated by recruitment and activation of caspases. Caspase-3 plays a dominant role in the hallmark caspase cascade which is the characteristic of the apoptotic pathway. The methanol extract (100 *μ*g/mL) treatment for 12 h caused a significant (*P* < 0.05) increase in caspase-3 activation of 1.8-fold, which was further increased to 2.4-fold compared to the control cells (Figure 3). Activation of caspase-3 is significantly increased by the methanol extract compared to the drug, sorafenib.

### 3.5. The Effects on Mitochondrial Membrane Potential (MMP)

**Figure 4:**
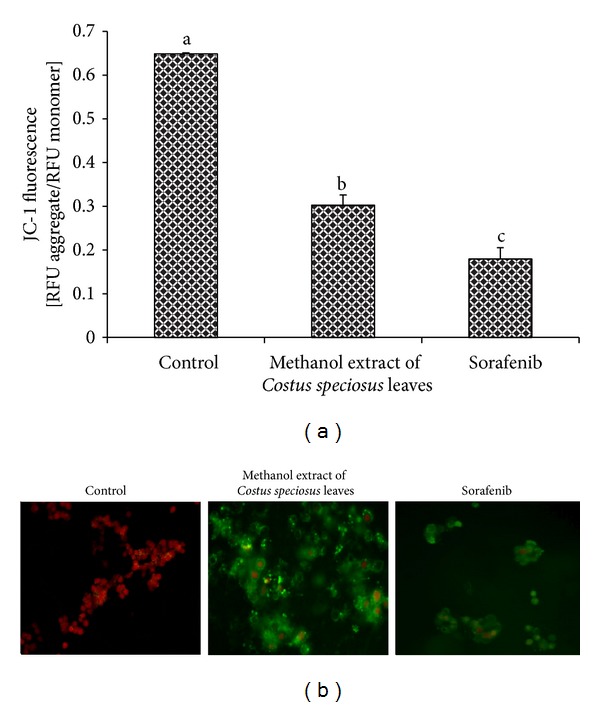
Effect of methanolic extract of* Costus speciosus* leaves on mitochondrial membrane potential (ΔΨ*m*). (a) Mitochondrial membrane potential (ΔΨ*m*) was measured by JC-1 fluorescence [fluorescence of JC-1 monomers (em 535 nm)/aggregates (em 590 nm)] in HepG2 cells treated with 100 *μ*g/mL of methanol extract or sorafenib for 24 h. Methanol extract or sorafenib showed significant decrease in ΔΨ*m* in HepG2 cells. Data are expressed as mean ± SD (*n* = 3). Columns not sharing the same superscript letter differ significantly (*P* < 0.05%). (b) Untreated control cells showed that cells have higher mitochondrial ΔΨ*m* as they are abundant in J-aggregates which is indicated by intense red fluorescence; methanol extract or sorafenib treated cells showed lower mitochondrial ΔΨ*m* which is represented by monomeric JC-1 with green fluorescence.

The mitochondrial membrane potential (ΔΨ*m*) is a reliable indicator of mitochondrial-dependent apoptosis and which can be quantified by applying the fluorescent dye JC-1. Treatment of the cells with the methanol extract or sorafenib at 100 *μ*g/mL for 24 h induced a reduction in mitochondrial membrane potential (Δ*ψm* depolarization), expressed as the reductions in JC-1 590/530 nm fluorescence ratios (0.648 ± 0.002 in control versus 0.302 ± 0.023 in cells treated with the methanol extract (*P* < 0.05) and 0.179 ± 0.026 in cells treated with sorafenib (*P* < 0.05) (Figure 4(a)). Cells treated with methanol extract or sorafenib showed green fluorescence indicating apoptotic cells with low mitochondrial Δ*ψm* whereas untreated control cells showed healthy with high mitochondrial Δ*ψm* with intense red fluorescence (Figure 4(b)). This reduction in mitochondrial membrane potential probably initiated the apoptotic cascade in the methanol extract treated cells.

### 3.6. The Effect on Cell Cycle Distribution

**Figure 5:**
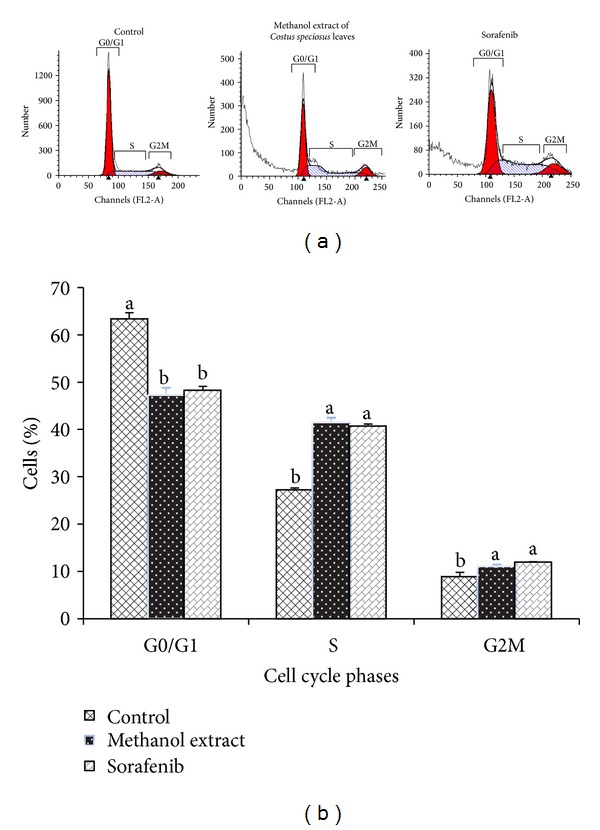
Effect of methanolic extract of* Costus speciosus* leaves on cell cycle distribution. Methanolic extract or sorafenib treatment significantly increased the proportion of S phase while significant decrease of G1/G0 phase. (a) Cell cycle analysis by flow cytometry. After exposure of HepG2 cells to 100 *μ*g/mL of the methanol extract or sorafenib for 24 h, cells were harvested, stained with propidium iodide, and analyzed by flow cytometry. Flow cytometric histograms are representative of two separate experiments. (b) Percentage of cell populations. The data are from one of two independent experiments with similar results. Data are expressed as mean ± SD (*n* = 3). Columns not sharing the same superscript letter differ significantly (*P* < 0.05%).

Inhibition of proliferation was further examined by measuring cell cycle distribution. At 24 h of treatment with the methanol extract (100 *μ*g/mL) or sorafenib (100 *μ*g/mL) or control cells with vehicle (<0.5% DMSO), an increase in S phase and G2/M phase (*P* < 0.05) concomitant with a decrease in G0/G1 phase (*P* < 0.05) was observed (Figure 5). The data indicates that the methanol extract mediated growth inhibition of HepG2 cells is associated with S and G2/M phase cell cycle arrest.

## 4. Discussion

Conventional chemotherapy remains ineffective in curing hepatocellular carcinoma due to its high hepatotoxicity. Several plant-based extracts have been shown to be effective in liver cancer therapy and prevention [16]. Essentially, the beneficial effects of plant extracts are due to their constituent phytochemicals that include polyphenols, carotenoids, alkaloids, and nitrogen and sulfur containing compounds [17]. Phytochemicals extracted from plants are excellent chemotherapeutic and chemopreventive agents which are well tolerated, nontoxic, easily available, and inexpensive [18].

*C. speciosus*, an ornamental plant, possesses diverse number of pharmacological activities and has long been used in traditional systems of medicine in many countries. Given the several health promoting attributes to* C. speciosus*, the principle objective of the study was to evaluate and establish the anticancer efficacy of* C. speciosus* leaf extracts using human hepatocellular carcinoma cell lines. Previous work demonstrated in* C. speciosus* rhizome extract exhibited antioxidant and antiproliferative properties in human cancer cell lines [15]. Methanol extracts of* C. speciosus* showed significant anti-inflammatory, analgesic, and antipyretic activities in experimental animals [19]. Our study represents the first report on anticancer effect of* C. speciosus* leaf extract in liver cancer cells.

We extracted phytochemicals from* C. speciosus* leaves using two different solvents: methanol and hexane. These solvents possess different polarity index values (methanol 5.1 and hexane 0.1) and have been used to extract a wide range of phytochemicals. By varying concentrations of the* C. speciosus* leaf extract, we found that the methanol extract was most effective in inhibiting growth of HepG2 cells in a dose- and time-dependent manner. Hexane extract did not show any notable antiproliferative effect in these cell lines, at any of the concentrations tested. Methanol with moderate polarity index is able to extract more efficacious constituents from the* C. speciosus* leaves than hexane with low polarity index which reflects the efficiency of methanol extract. The observed inhibitory activities are the results of the biologically active constituents extractable by solvent, efficiency, and polarity of index of each extracting solvent.

Selective induction of apoptosis is a highly desirable trait in ideal chemopreventive and chemotherapeutic regimens. The methanol extract efficiently induces apoptosis in HepG2 as determined by Annexin V/7-AAD assay and activation of caspase-3. The decreased mitochondrial membrane potential by the methanol extract treated cells demonstrated that mitochondrial apoptotic pathway occurred by the opening of mitochondrial permeability transition pore. The methanol extract induced apoptosis is largely mitochondria mediated and associated with the collapse of the transmembrane potential which results in the expulsion of key apoptogenic molecules such as cytochrome *C* from the mitochondria. Our study shows remarkable inhibition of cell proliferation by the methanol extract with proapoptotic effects together with cell cycle arrest at S and G2/M phase.

Herbal and traditional plant medicines emerged as the highest antioxidant-containing products in various studies [20]. Antioxidant rich plant medicines significantly reduce the risk of many cancer diseases suggesting that antioxidants could be effective agents for the inhibition of cancer spread [21]. Total polyphenol content of methanol extract of* C. speciosus* leaves is 11.45 mg/g (expressed as gallic acid equivalents) [22]. The antioxidant activity of methanol extract of leaf was demonstrated by its higher hydroxyl radical scavenging activity and free radical quenching ability [22]. Diosgenin is the major constituent isolated from* C. speciosus* [23] with maximum quantity of 0.37% in leaves (Srivastava et al., 2011). Diosgenin has the antiproliferative property by strongly generating ROS and this oxidative stress induces apoptosis in HepG2 cells through activation of JNK/p38 MAPK pathway [24]. The antioxidant phytochemicals including diosgenin may exert the antiproliferative effect to methanol extract of* C. speciosus* leaves which need further investigation.

In conclusion, our current study is the first to identify the remarkable anticancer activity of methanol extract of* C. speciosus* leaves in liver cancer. Our data generate compelling evidence for further evaluation of this extract and constituent phytochemicals as chemopreventive regimen for liver cancer. Studies to determine the composition of the extract, identification of active principle, and its effect on critical genes associated with liver cancer are underway. These studies are valuable for identifying plant-based chemotherapeutic drugs as currently used liver cancer drug sorafenib causes many side effects. However, animal and human studies are indeed required to prove the safety and efficacy of application of leaf extract of* C. speciosus* as a potential chemopreventive agent in clinical practice.

## Figures and Tables

**Table 1:** Cytotoxic effect of various doses of methanol and hexane leaf extracts of *Costus speciosus* or sorafenib for 24 h and 48 h incubations.

Source	Concentration	% viability	
		24 h	48 h
Methanol extract	Control	100.0 ± 7.2^a^	100.0 ± 11.1^a^
	1 *μ*g/mL	100.7 ± 0.7^a^	100.9 ± 0.6^a^
	10 *μ*g/mL	102.1 ± 1.1^a^	98.8 ± 5.4^a^
	50 *μ*g/mL	89.6 ± 3.4^b^	86.2 ± 4.7^b^
	100 *μ*g/mL	43.9 ± 2.5^c^	32.1 ± 4.9^c^
	200 *μ*g/mL	7.3 ± 0.3^d^	6.4 ± 0.1^d^

Hexane extract	Control	100.0 ± 7.2^a^	ND
	1 *μ*g/mL	99.8 ± 2.9^a^	ND
	10 *μ*g/mL	99.7 ± 5.1^a^	ND
	50 *μ*g/mL	99.3 ± 2.9^a^	ND
	100 *μ*g/mL	89.9 ± 5.5^a^	ND
	200 *μ*g/mL	84.9 ± 8.4^a^	ND

Sorafenib	Control	100.0 ± 7.2^a^	100.0 ± 11.1^a^
	1 *μ*g/mL	74.1 ± 6.1^b^	27.7 ± 2.1^b^
	10 *μ*g/mL	30.3 ± 2.9^c^	22.6 ± 1.2^b^
	50 *μ*g/mL	10.1 ± 0.9^d^	5.5 ± 0.2^c^
	100 *μ*g/mL	7.4 ± 0.9^d^	1.0 ± 0.4^c^
	200 *μ*g/mL	3.6 ± 0.7^d^	0.8 ± 0.6^c^

The percentage of cell viability was measured by MTS assay. Data are presented as mean ± SD of three replicates from three independent experiments. Values that are not sharing the same superscript letter differ significantly (*P* < 0.05) compared to control. NA: not applicable; ND: not determined due to no effect

**Table 2:** Cytotoxic effect of methanol extract of *Costus speciosus* or cancer drugs (positive control) on cancer and normal cells after 24 h and 48 h of exposure.

	Cytotoxicity assay IC 50 (*μ*g/mL)					
Test compounds	Human hepatocellular carcinoma HepG2 cells		Human acute monocytic leukemia cells THP-1		Normal lung cells	
	24 h	48 h	24 h	48 h	24 h	48 h
Methanol extract of *Costus speciosus *leaves	93.3	77.3	58.1	42.2	>100	>100
Sorafenib (liver cancer drug)	3.8	0.4	—	—	—	—
Daunorubicin hydrochloride (leukemia drug)	—	—	0.6	0.4	—	—

## References

[1] Davis GL, Dempster J, Meler JD (2008). Hepatocellular carcinoma: management of an increasingly common problem. *Proceedings of Baylor University Medical Center*.

[2] Pang R, Tse E, Poon RTP (2006). Molecular pathways in hepatocellular carcinoma. *Cancer Letters*.

[3] Mittal S, El-Serag HB (2013). Epidemiology of hepatocellular carcinoma: consider the population. *Journal of Cinical Gastroenterology*.

[4] Takayama T, Makuuchi M, Hirohashi S (1998). Early hepatocellular carcinoma as an entity with a high rate of surgical cure. *Hepatology*.

[5] Poon RT, Fan ST (2004). Resection prior to liver transplantation for hepatocellular carcinoma: a strategy of optimizing the role of resection and transplantation in cirrhotic patients with preserved liver function. *Liver Transplantation*.

[6] Garrean S, Hering J, Saied A, Helton WS, Espat NJ (2008). Radiofrequency ablation of primary and metastatic liver tumors: a critical review of the literature. *American Journal of Surgery*.

[7] Lencioni R (2012). Chemoembolization for hepatocellular carcinoma. *Seminars in Oncology*.

[8] Horgan AM, Dawson LA, Swaminath A, Knox JJ (2012). Sorafenib and radiation therapy for the treatment of advanced hepatocellular carcinoma. *Journal of Gastrointestinal Cancer*.

[9] Furuse J (2008). Sorafenib for the treatment of unresectable hepatocellular carcinoma. *Biologics: Targets and Therapy*.

[10] Qurishi Y, Hamid A, Majeed R (2011). Interaction of natural products with cell survival and signaling pathways in the biochemical elucidation of drug targets in cancer. *Future Oncology*.

[11] Srivastava S, Singh P, Jha KK, Misha G, Srivastava S, Khosa RL (2012). Evaluation of anti-arthitic potential of the methanolic extract of the aerial parts of *Costus speciosus*. *Journal of Ayurveda and Integrative Medicine*.

[12] Bavarva JH, Narasimhacharya AVRL (2008). Antihyperglycemic and hypolipidemic effects of *Costus speciosus* in alloxan induced diabetic rats. *Phytotherapy Research*.

[13] Liu M-J, Wang Z, Ju Y, Wong RN-S, Wu Q-Y (2005). Diosgenin induces cell cycle arrest and apoptosis in human leukemia K562 cells with the disruption of Ca^2+^ homeostasis. *Cancer Chemotherapy and Pharmacology*.

[14] Anuchapreeda S, Tima S, Duangrat C, Limtrakul P (2008). Effect of pure curcumin, demethoxycurcumin, and bisdemethoxycurcumin on WT1 gene expression in leukemic cell lines. *Cancer Chemotherapy and Pharmacology*.

[15] Baskar AA, Al Numair KS, Alsaif MA, Ignacimuthu S (2012). In vitro antioxidant and antiproliferative potential of medicinal plants used in traditional Indian medicine to treat cancer. *Redox Report*.

[16] Stagos D, Amougias GD, Matakos A, Spyrou A, Tsatsakis AM, Kouretas D (2012). Chemoprevention of liver cancer by plant polyphenols. *Food and Chemical Toxicology*.

[17] Karna P, Gundala SR, Gupta MV (2011). Polyphenol-rich sweet potato greens extract inhibits proliferation and induces apoptosis in prostate cancer cells in vitro and in vivo. *Carcinogenesis*.

[18] Sak K (2012). Chemotherapy and dietary phytochemical agents. *Chemotherapy Research and Practice*.

[19] Srivastava S, Singh P, Jha KK, Mishra G, Srivastava S, Khosa RL (2011). Anthelmintic activity of aerial parts of *Costus speciosus*. *International Journal of Green Pharmacy*.

[20] Paur I, Carlsen MH, Halvorsen BL, Blomhoff R, Benzie IFF, Wachtel-Galor S (2011). Chapter 2. Antioxidants in herbs and spices: roles in oxidative stress and redox signaling. *Herbal Medicine: Biomolecular and Clinical Aspects*.

[21] Deep G, Dhiman M, Rao AR, Kale RK (2005). Chemopreventive potential of Triphala (a composite Indian rug) on benzo(a)pyrene induced forestomach tumorigenesis in murine tumor model system. *Journal of Experimental & Clinical Cancer Research*.

[22] Vijayalakshmi MA, Sarada NC (2008). Screening of *Costus speciosus* extracts for antioxidant activity. *Fitoterapia*.

[23] Dasgupta B, Pandey VB (1970). A new indian source of diosgenin (*Costus speciosus*). *Experientia*.

[24] Kim DS, Jeon BK, Lee YE, Woo WH, Mun YJ (2012). Diosgenin induces apoptosis in HepG2 cells though generation of reactive Oxygen species and mitochondrial pathway. *Evidence-Based Complementary and Alternative Medicine*.

